# Patient and public involvement, engagement, and participation in practice: co-production of a creative health approach and theory of change through the ReCITE consortium-building project in Liverpool

**DOI:** 10.1186/s40900-026-00846-z

**Published:** 2026-02-26

**Authors:** Dawn Holford, Charlotte Hemingway, Kim Ozano, Amina Ismail, Sarah Glover, Sarah Maclennan, David Lewis, Mike Morris, Madeline Heneghan, Aidan Jolly, Victoria I. Ekpo, Nour Essale, Reda Madroumi, ReCITE Consortium, Miriam Taegtmeyer, Rachel Tolhurst, Gillian Kyalo, Amina Ismail, Mike Morris, Natalie Denny, Amanda Fulford, Sarah Maclennan, Victoria Ekpo, Vicki Doyle, Kim Ozano, Sarah Glover, Rachel Tolhurst, Miriam Taegtmeyer

**Affiliations:** 1 University of Bristol, Bristol, UK; 2 Liverpool School of Tropical Medicine, Liverpool, UK; 3 The Stop, Collaborate and Listen Agency Ltd, Wrexham, UK; 4 Liverpool John Moores University, Liverpool, UK; 5 Central Liverpool Primary Care Network, Liverpool, UK; 6 Writing on the Wall, Liverpool, UK; 7 Collective Encounters Theatre for Social Change, Liverpool, UK; 8 Edge Hill University, Liverpool, UK

**Keywords:** Co-production, Public engagement, Creative health, Community involvement, Health equity

## Abstract

This paper describes a process of working together to design a research programme that explores how creative activities such as storytelling could help reduce health inequalities. This work was part of a project called ReCITE, which brought together people from community organisations, health services, the arts, and universities in Liverpool. Fifty-six different people took part in six workshops over nine months. Together, they discussed how creative projects could support people’s well-being and make health outcomes fairer. From these discussions, the group developed a shared plan (known as a theory of change) showing how creative activities might lead to better health for everyone. This plan also helped design a research programme, which later received funding to continue the work. The process involved several rounds of gathering feedback, revising ideas, and returning to participants to check that their views were represented. Working together over time helped build trust and shared ownership of the research. However, it was sometimes difficult for community partners to stay involved throughout the process because there was limited funding to support their time and participation.

**Supplementary Information:**

The online version contains supplementary material available at 10.1186/s40900-026-00846-z.

## Abstract (structured)

**Background:**

Co-production with public stakeholders is increasingly recognised as an important approach to ensuring health research is relevant to communities and interventions and likely to generate sustainable change. Ideally, co-production needs to be sustained throughout different stages of research and allow stakeholders ownership of the research agenda. We explore a case study of iterative co-production conducted over a nine-month period with a wide range of disparate stakeholders that transformed into a research-ready consortium able to design and obtain funding for a creative health research programme.

**Main body:**

The ReCITE consortium is comprised of academics, creatives, and specialists in capacity development and community engagement in Liverpool, UK. Its main focus is health equity and coming up with new ways of working to tackle entrenched, avoidable and unfair differences in health. ReCITE led a series of six workshops in 2023 to co-produce a research programme to investigate creative approaches for improving health equity. Fifty-six stakeholders from the academic, creative and arts, health, community and voluntary sectors participated across six workshops that sought broad engagement and consolidated stakeholder input to develop a research agenda and expand the consortium. Common emerging themes were: (1) understanding complexities in funding creative health; (2) investigating storytelling as a catalyst for change; (3) achieving change through collective action; (4) the role of creative advocacy to change societal and funding structures; (5) evidencing the impact of community-led creative health interventions on health equity. These formed multiple co-produced outputs, including five pillars of a theory of change for the research that underpinned the research questions and determined five interventions to take forward by the research-ready consortium to securing funding for future work.

**Conclusion:**

The iterative process of gaining stakeholder input, consolidating it, and seeking further input, helped to incorporate the perspectives of different stakeholder groups into different project outputs. Equitable power-sharing guided decision-making over what to prioritise was important in building ownership of the research agenda and trust among stakeholders. However, this was a lengthy iterative process and sustaining time commitment was difficult for those stakeholders who were not fully funded to participate.

**Supplementary Information:**

The online version contains supplementary material available at 10.1186/s40900-026-00846-z.

Patient and public involvement, engagement, and participation (PPIEP) in research have become increasingly embedded in health research, with institutions, funders, and researchers recognising it is an important step for research relevance [1]. A common approach to public and patient involvement is co-production, which represents a broad set of processes to engage public stakeholders at distinct stages of health research or in improving healthcare systems [1–3]. In terms of the different possible levels of PPIEP (ranging from informing the public to empowering the public, e.g., [4]), co-production operates at the higher levels of engagement, as it aims to achieve sustained dialogue between researchers and stakeholders, participatory decision-making, and joint ownership of outputs [5]. Therefore, co-production should be a dynamic process that is sustained over time through a variety of engagement methods, interactions, and social relations [6]. However, this contrasts with most research papers and case studies that report on co-production of a specific study design or output [1].

This paper presents a case study of co-production with two novel perspectives. First, the aim of the co-production work was to develop and sustain a community-led creative health approach to addressing health inequity. Creative health refers to a range of creative activities that benefit holistic health and wellbeing, including supporting health and care service [7]. While creative activities are commonly used as methods for PPIEP [8, 9], they are less common as the goal of the co-production exercise. Second, the engagement process itself (i.e., how to go about the co-production work, the bonding of a team ready to take the work forward) was as much an output of co-production as tangible outputs such as strategies, publications, and funding applications. A key objective was to address complex health problems and inequities in communities and understand how best to sustain a community-led creative health approach to doing so. Creative approaches are already used to collect and understand data on community health inequalities, strengthen agency and solidarity among individuals and communities, and communicate messages across groups (e.g., health messages from authorities to communities; experiences of inequity from communities to policy-makers [10]). However, a focus on evaluating creative health as a means to improving health equity is still relatively new. The extant literature has a large focus on its impact on individual health outcomes, and there is still very little evidence of its potential to tackle health inequity and its social determinants [11–13].

## The co-production process

**Fig. 1:**
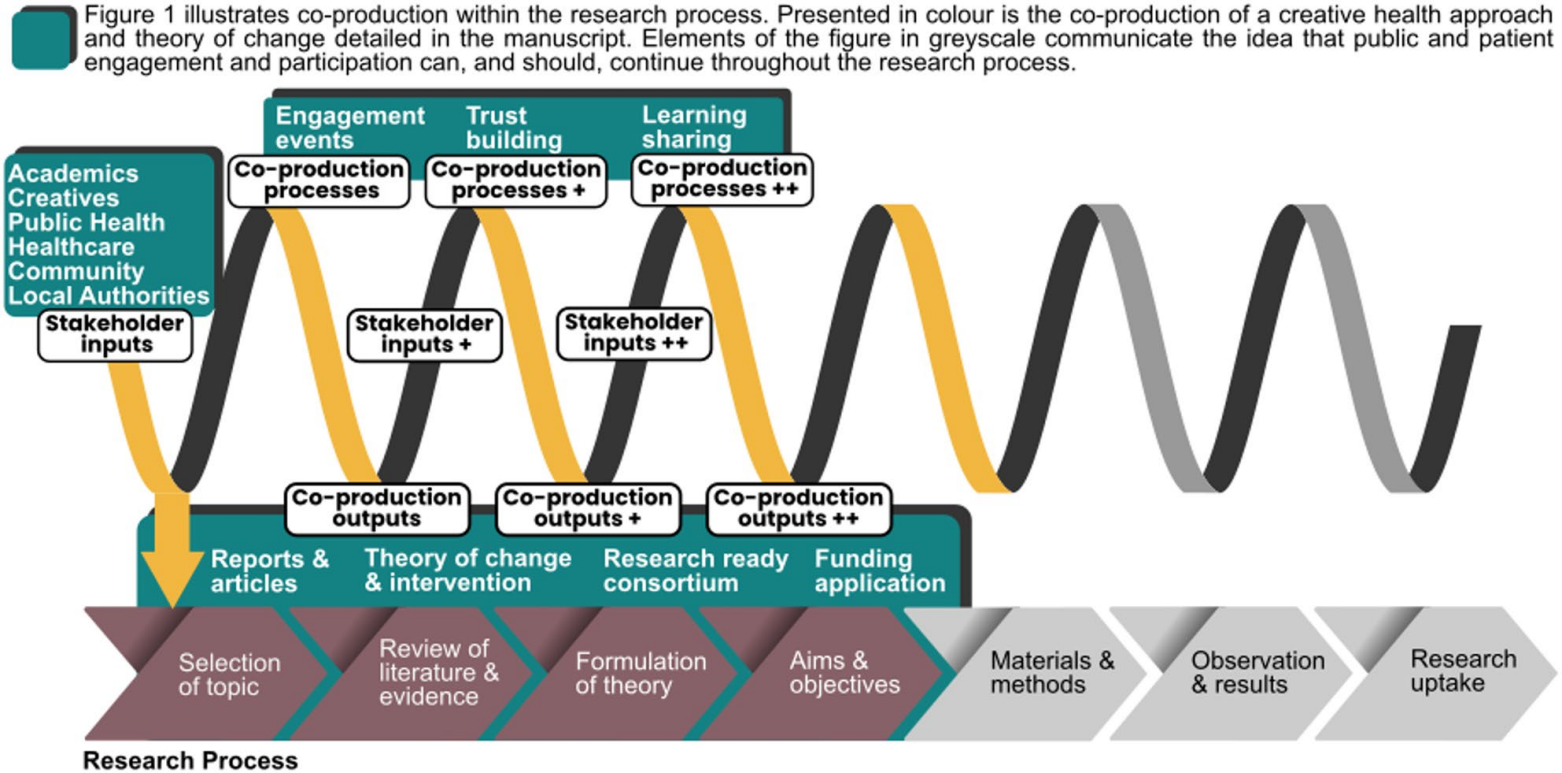
Illustration of the iterative co-production process

These insights on the potential for creative approaches to improve health equity framed co-production activities and the goals and outputs reported in this paper. Reflections are presented on an iterative process where initial input from all stakeholders shaped the co-production process and manifested in outputs, which themselves were reviewed by initial and new stakeholders and fed forward into new inputs, later processes and outputs (see Fig. 1). The following sections describe the background of the project and its context, explain the co-production methodology and its outputs (see Table 1 for a summary), and analyse how a theory of change was drawn out of the emerging themes.

As shown in Fig. 1, the outputs of co-production refer to a variety of products with different contributors at different times. This paper will focus on the theory of change as a focal example as its main contribution to the literature, as it provides a model that others can build on for investigating how creative health can support communities, through the medium of storytelling, to lead interventions that address health inequity. The methodology of producing the theory of change through workshops that iteratively consult and consolidate stakeholder input forms a second contribution, as a model for co-production that may be applied in future participatory research.

### The ReCITE project: Building research by communities to address inequalities through expression

The ReCITE project started as a collaborative effort of an initial group based at universities (Liverpool School of Tropical Medicine, Edge Hill University, and Liverpool John Moores University), a creative organisation (Writing on the Wall), and capacity development specialists (Capacity Development International and the Stop, Collaborate and Listen Agency). Over time and through the workshops described in this paper, it expanded into a wider partnership. Its original overarching goal was to build a research-ready consortium in Liverpool that would bring together academics, arts organisations, health providers and community groups: those with active investment and interest in using creative approaches to improve people’s health in Liverpool’s most underserved communities. In this context, “research-ready” meant not just having the relevant expertise and experience within the consortium, but also an understanding of the research context, defined and agreed research questions to investigate the impact of creative health approaches, and a trusted relationship among team members to undertake research. To achieve this, the consortium planned to (1) map community and arts organisations in Liverpool involved in creative health; (2) review approaches to evaluate their interventions; (3) strengthen capacity of stakeholders to co-produce research questions and programmes; (4) co-produce a theory of change and related research programme for a community-led creative health approach to tackling avoidable and unfair differences in health.

Our focus was on Liverpool, a city with a vibrant ecosystem of third sector and arts organisations engaged with diverse communities, but with stark health inequities exacerbated by the Covid-19 pandemic. Life expectancy within Liverpool is approximately 3 years below the national average and differs by more than 12 years between the most and least deprived wards [14]. Government cuts to local authority spending have hit Liverpool’s creative and community sectors, contributing to a vicious cycle in which lack of funding creates competition for small amounts that limits the reach of community-based arts activities and undermines collaborative working. This in turn, limits evidence of impact to justify larger-scale and more sustained funding.

### Positionality statement

This paper is one of the outputs of the ReCITE consortium. It reflects the perspectives of a range of participants in the workshop, including original consortium members involved from the outset and members who joined later. As co-authors, we represent the academic, creative, health, and capacity development sectors based on our professional affiliations. However, many of us also have lived experience as members of communities in Liverpool and have worked extensively with them over the years.

## Methods

Co-production activities took place over six face-to-face workshops between December 2022 and May 2023. We used these workshops to bring together stakeholders from different sectors to share existing knowledge and jointly develop new knowledge to inform our goal of co-producing a creative health theory of change and research approach using a range of methods. Multiple workshops allowed these outputs to develop iteratively over time, while also facilitating sustained engagement from participants that enabled the building of trusted relationships among the consortium and new members, supporting its transformation into a research-ready consortium.

### Participants

Fifty-six unique participants attended the workshops. Participant recruitment built on the long-standing trusted relationships and reputation of the consortium partners with different sectors to ensure broad representation of key stakeholders. Table 2 shows the breakdown of participants by sector and how this was spread across time (sustained engagement) and workshops. Because the project focused on health equity, involvement of “patients and the public” is extremely broad, covering residents of underserved communities in the target regions—who all have a stake in health and public health services, but may also have dual roles working in community, creative, and health organisations within or supporting those communities. For this reason, we describe participants using the term “stakeholders”, reflecting the large stake they have in work that addresses health equity in Liverpool.

Participants were recruited using stakeholder mapping and snowball sampling to identify individuals from organisations with known interests, skills, and/or lived experiences related to arts and culture initiatives, health disparities, community outreach, and research.

In Table 2, *researchers* include academics and research institution staff working in disciplines related to public health and creative arts. *Creative/Arts sector* includes representatives from arts and culture organisations (e.g., museums, theatres) or who had experience delivering artistic projects for or with impacts on community health and wellbeing (e.g., creative writing). *Community sector* includes representatives from community, voluntary, and faith organisations that were embedded in and supported communities in Liverpool, particularly in the most deprived areas. *Health sector* includes representatives working in public health (e.g., from local authorities) and in the health service (e.g., from primary and secondary care). *Capacity development* includes experts in facilitating workshops and building research capacity. Many participants held dual roles, with voluntary positions in creative and arts organisations as well as in the community sector.

Most consortium members were representatives of their sector who had personal lived experiences related to health disadvantages and professional experiences of working with and/or supporting others facing health disadvantage. Focal health outcomes for discussion were not specified, leaving this to be community-led. This introduced some complexity into the meaning of “lived experience” in our context of health inequity. The lived experiences heard from in this project varied widely and included unfair experiences based on individuals’ position along different axes of disadvantage, such as race/ethnicity, disability, geography, and intersections of these. For example, health disparities for participants from Gypsy Traveller communities in Liverpool stem from an intersection of minority status, impermanent residence, and discriminatory assumptions made by health providers about their health statuses, compounding disadvantages in access to care.

### Workshops

Each consortium-building workshop had a theme for discussion (see Table 1) following a journey of bringing people together and turning ideas into actions. The workshop plan interspersed broad-based discussions with larger groups and wider representation (workshops 1, 3, 5) with action-specific activity to consolidate and move forward ideas towards outputs (e.g., producing the theory of change; workshops 2, 4, 6). Lived experience was explored in the broad-based discussion by inviting people to share and/or present experiences as part of the workshops. In the consolidation workshops, although exploring lived experience was not formally included in the format, participants often volunteered their personal experiences on the topics discussed.

The initial workshop and the overall structure was designed by the original ReCITE consortium partners, but attendees at each subsequent workshop helped to shape content for the next. To support non-academic partners in contributing to workshops focused on developing research, the team’s capacity development specialists ran a parallel series of three training sessions in community-based participatory research methods.

Skilled facilitators from the consortium led the workshops using a range of creative methodologies for participatory input (see Table 1). Facilitators drew on their experience of conducting participatory action research to use methods allowing all voices to contribute. They encouraged creative stakeholders to add impromptu exercises to illustrate points, raise energy in the room or enable open discussion. Reports were then shared with all participants for their review and to inform decisions about content and methods for later workshops. Key facilitators met between workshops in smaller subgroups to review and reflect on inputs and synthesise learning to prepare for facilitating subsequent workshops.

### Data capture and analysis

Workshop discussions and their associated textual and visual outputs were documented in real time by note-takers to include in a workshop report. Workshop reports were produced by the capacity development partners. Each workshop report summarised the objectives and outputs, methods, and key discussion points and actions from each activity. Transcribed notes and visual and textual documentation were appended to the workshop reports to enable capture of the full range of contributions. These were captured anonymously to encourage free expression from all participants during the workshops. Participants were encouraged not just to speak their views but also write them on post-it notes. Analysis of the workshop reports and preparation of this academic paper was led by the academic team. The co-authorship team (affiliations and backgrounds as described in the authors’ list and positionality statement) agreed to the interpretation and conclusions discussed. Illustrative quotes reported in this paper were transcribed verbatim from the textual documentation.

**Table 1:** Objectives, methods and outputs in each of the recite workshops

Workshop	Objectives	Methods	Outputs
1: Structures and processes	Conceptualisation, planning, and introduction Identification of additional stakeholders	Discussion exploring motivations for joining the project. Plenary discussions for a theory of change. Presentations and discussions to conceptualise consortium structures. Individual brainstorming on consortium structures and the theory of change. Manual doodle to plan subsequent workshops Stakeholder mapping	Workshop plan Stakeholder map Scope of rapid literature review
2: Rapid literature review and theory of change	Explore areas of potential impact for research and pathways to change Begin to develop theory of change Design next workshop	Presentation and plenary discussion of rapid literature review Group work and brainstorming on theory of change and desired research programme.	Working group for theory of change development
3: Arts, health and community group perspectives	Map creative health interventions in Liverpool and how those are used in and by communities Understand health equity priorities of communities Expand understanding of arts commissioning and prescribing for health and health equity	Rapid 2-min presentations from community and arts-based groups. Plenary discussions. Multidisciplinary group work and brainstorming about health equity priorities. Panel discussion with health commissioners and providers. Presentations on.	Identified health equity priorities for research programme Plans for developing a theory of change.
4: Theory of change and research priorities	Development of theory and change and research priorities	Presentations on incorporating previous workshop insights into a project plan and theory of change. Discussions and collaborative analysis of priorities identified in workshop 3.	Draft theory of change Plan for participatory action research training
5: Programme design	Understand a community-led approach to tackle health inequity and envision change using a creative health approach	Presentation of a community-led model. Plenary discussion around research programme design. Visioning exercise for impact of a research programme.	Refined theory of change Draft research questions and programme Bespoke participatory action research training
6: Research questions	Summarise consortium thinking Review a research funding call and prepare a funding bid. Refine research questions, priorities, and focus of the research programme	Presentation on consortium’s work, funding opportunity, and relevant academic work. Plenary discussion around the funding call and shaping the research programme.	Theory of change – logic model and implementation plan Research questions and programme Outline for a funding bid

**Table 2:** Breakdown of participants at each recite workshop

Workshop	Number of participants						
	Total	Academic	Creative/ Arts sector	Community sector	Health sector	Capacity development	Engaged in a previous workshop
1: Structures and processes	29	9	9	1	7	3	-
2: Literature review and theory of change	12	4	3	0	1	4	10
3: Arts, health and community group perspectives	34	6	12	7	6	3	14
4: Theory of change and research priorities	10	3	2	0	1	4	10
5: Programme design	17	5	6	2	1	3	15
6: Research questions	14	5	3	1	1	4	13

Note. Classification is based on participants’ self-identified primary affiliation whose interest they represented at the workshops. Some participants held dual affiliations (e.g., academic in a creative or health field, community mobiliser employed by a university). The total number of unique participants was 56 (see Table 3)

**Table 3:** Breakdown of unique and repeat attendance at workshops

	Participants	*Organisations*				
		Academic	Creative	Capacity Development	Community	Health
Unique attendees	56	10	21	4	9	12
Number who attended all 6 workshops	6	2	1	2	0	0
Most workshops attended for an organisation in sector	-	6	6	6	3	3

## Emerging workshop themes

In this section, we present emerging themes from workshop discussions and demonstrate how they fed into subsequent discussions and outputs. We map themes from discussions across workshops to their representation in ReCITE’s theory of change, showing how they shaped it.

### Workshop 1: Structures and processes

Workshop 1 initiated introductions and discussions across stakeholders representing the relevant creative, health, community, and academic sectors to set the direction for building the consortium and developing its outputs. Participants discussed their motivations for the work, how they envisioned collaborating as a consortium, and what pathways to change ReCITE should tackle in a research programme. A core motivation for involvement in the consortium was members’ desire to see change for communities of Liverpool, particularly in influencing policy. Speaking from their own experiences in disadvantaged communities, they spoke of the many capabilities and core assets already within those communities that could support health equity, but described a sense of jadedness when decisions and resource allocation continually disregarded or undervalued community expertise.

[We should] look at the things communities are already doing well. Communities have everything they need in terms of power [to support their members] but they are not given the resources.

Participants felt the right stakeholders needed to be brought in to shape the research vision for change, especially those who worked at the intersection of health and community. This led to a stakeholder mapping exercise that informed subsequent recruitment to workshops and to join the consortium. Three broad set of values underpinned discussions of how the consortium should work together:

1) Inclusivity, which encompassed accessibility, respect for but also celebration of diverse contributions and expertise, and should emphasise listening and learning from one another;

[We should be] open to all voices and what they may be trying to express.

2) Meaningful co-production, which was expressed in terms of valuing the expertise in communities and of each person contributing to ReCITE, while also making sure contributions lead to actionable outcomes;

[We should value] learning from each other and creating new knowledge for change/action.

3) A commitment to social justice, where knowledge is created for change and action. This encapsulated efforts to tackle the complex social determinants of health inequity and challenge existing thinking, paths of influence, and power structures.

What drives me is storytelling for social justice. Voice has power and their [communities’] story matters.

All workshop participants agreed that a creative health approach would provide strong health benefits to individuals, especially those with complex health needs. In particular, participating in storytelling (in various artistic forms including written, poetry, audiovisual etc.) was highlighted as a more accessible—and thus “democratic”—way for individuals to share their health experience and spotlight unfair and avoidable differences that communities faced:

Arts [through creativity and storytelling] provides a democratic means of accessing the conversation about our health and wellbeing.

They spoke of equity gaps in their communities showing up in the health stories they were exposed to, which framed individuals’ expectations and outcomes. One stakeholder described their experience of how communities differed in how they viewed cancer, based on the experiential stories of people in those communities:

If you live in [a] poor community then you know people with late diagnosis, vs. if you live in a rich community you will know someone who survived.

These insights were taken forward into a rapid literature review^1^ to assess existing evidence for creative health approaches and the ways it could lead to changing health outcomes.

### Workshop 2: Rapid literature review and theory of change

Workshop 2 discussed evidence from the rapid literature review, the scope and search terms for which were based on inputs from Workshop 1. Participants used the findings to shape planning a community-led creative health approach and how interventions could impact health equity at the level of individuals, communities, and society.

In their discussions, participants reflected that a lot of the work captured in the literature review focused on the impact of creative approaches on health and wellbeing at the individual level. They were interested in whether creative health could have a more sustained systemic impact on health equity but noted that although some reports claimed that this might be possible (e.g., [11]), there was little evidence yet to support it [13].

Of interest to participants were ways in which storytelling narratives could be used to challenge societal discourses contributing to health inequities, raise awareness of health and wellbeing challenges facing disadvantaged people, and advocate for structural changes to address these. For example, they reflected on several description reviews of how narratives might promote health and influence policy [15–17].

At the same time, participants picked up on three challenges to wider implementation of creative health approaches, which they recognised from their own experiences. First, third sector organisations are highly experienced in delivering arts and storytelling interventions in communities but are limited in their ability to conduct robust evaluation due to a lack of resources and skilled personnel [18]. Second, the evidence collected and reported for the health-related benefits of creative approaches tended to be short-term and focused on individuals, masking the potential to impact complex, population-level outcomes such as societal health equity [19]. Third, the literature lacked conceptual and theoretical frameworks to explain the mechanisms by which creative arts such as storytelling might benefit health [20].

[there is] so much focus [in the literature] on individual not structural elements that drive inequity.

Based on this analysis, participants felt the consortium would need to sustain co-production between third sector creative and community organisations and academics, each of whom had contributory expertise. Some thought that evidence would be stronger if it could be combined across sectors (though they did not discuss the practicalities of doing so).

Linking sectors/contexts to get a sense of evidence across different services/providers – get a larger meta-level evidence [sic] by linking smaller studies.

In addition, to fill the existing research gap, they would need to measure the impact of creative health interventions beyond individual-level benefits and try to tackle the mechanisms of realising population health changes:

Strengthened identity [leads to] increased confidence and capacity [leads to] social representation [leads to] critical consciousness [leads to] increased agency (empowerment)

Participants questioned whether they might need to advocate for the value of stories as evidential data:

*Whose quality? What does quality mean when looking at storytelling? …*

Storytelling is often seen as low value evidence in research.

Recognising that a research programme would be time-limited, participants discussed the areas that could be addressed and evidenced with creative health interventions. Hardship and mental health were priority areas for participants, and they agreed here to take a community-led approach to explore and address needs at the community level, while committing to promote advocacy for structural change.

### Workshop 3: Arts, health and community group perspectives

Workshop 3 picked up on the theme of exploring community needs and widened the discussion, building on stakeholder mapping from Workshop 1 to recruit a larger group of stakeholders, particularly from the community sector such as voluntary, charity, and social enterprise organisations (see Table 2). Space was given to these participants to share their lived experience from the communities they belonged to and served, especially their experiences of using creative methodologies to support the members of disadvantaged communities. This helped to gather diverse perspectives on community priorities for health equity and how storytelling might be used as a creative health approach to improve health equity. These perspectives shaped thinking and conceptualisation work taken forward in Workshops 4–6 to concretise the ReCITE research approach and theory of change.

***Community health equity priorities.*** There were cross-cutting themes in these group discussions of: improving access to care, building trust, creating platforms to share stories, challenging negative health narratives and misinformation, and how people’s mental health is intertwined with most health outcomes. Across these, there was a consensus that the focus and outcomes of any creative health intervention should be community-led, and had to be “preventative, positive, and long-term focused”, generating sustained funding that could continue to impact communities.

*The fundamental question is how can we create platforms that can be sustained and that will attract the attention of funding organisations? How can we bring people together?*

***Using storytelling to impact health equity.*** Participants expressed how storytelling, as a method in creative health, connected and empowered people, allowing them to share lived experiences and advocate for their communities. There was recognition that stories could generate an appetite for change, while on the flip side, they could act as a barrier to change by perpetuating fixed views or stereotypes of situations. One participant discussed how people in their community preferred to talk about how things *were* for them instead of how they *could be*:

*[It’s]* frustrating because we want to talk about the positives in our communities, but we often end up speaking about discrimination. … *Internal stories that are told within communities can maintain inequalities.*

To make a positive difference, participants felt stories needed to be told *for change*, i.e., in a way that invited people to look forward and imagine the future rather than as a tale of deficits:

Sometimes our young people don’t want to know more about how unequal society is. How do we flip it from a deficit? Where’s the empowerment and the joy?

A challenge around this was getting the right people to listen to community stories about unfair barriers that contributed to health disadvantages. As an example, a team of a Primary Care Network partnered with community organisations obtained funding to gather community insights, which they then used to design their MMR vaccination public health campaigns, shifting the focus to “immunisation” and emphasising the availability of a gelatine-free vaccine option that was acceptable for their communities. These insights were taken up by national campaigns, but there was then no further funding for them to properly utilise their rich community insights to effect change and further disseminate campaign materials locally. Team members felt this response to their efforts was disempowering:

*Our communities have rich narratives*,* and the small pockets of funding can be negative as it gives communities insight into inequities but doesn’t give people power. How do we get the people with the power to see this?*

Participants picked up on an earlier tension identified in Workshop 2, between evidence valued by decision-makers and evidence valued at the grassroots level. Health commissioners explained that it was their responsibility to use funds wisely and to secure evidence of value for money and of objective and measurable change. However, they did recognise objective data were not always possible to acquire, and subjective evidence did have to be considered:

Now more than ever commissioners are expected to show effective, efficient commissioning that is scalable and impactful, strong evidence is needed for this, also interventions that can tackle multiple outcomes will be favourable as value for money.

Representatives of community and arts organisations shared their experience of conducting social evaluations at the grassroots level that were not valued by health commissioners. They felt these should be considered by decision-makers and questioned why only some evidence—notably not “stories” that they collected—was accepted as robust:

Many [arts and community] organisations are collecting stories using social science approaches [such as ethnography and photovoice], but [these] are not positioned as data or ‘scientific’ evidence. What is needed to show decision-makers that stories are data [able to] shape health outcomes.

### Workshop 4: Theory of change and research priorities

Workshop 4 discussed and consolidated the thinking thus far into a draft theory of change and identified health equity priorities based on the perspectives gathered in Workshop 3. The focus was on a creative health approach able to catalyse change among communities, lead to collective action and community-led solutions, and embed evaluation to demonstrate the effectiveness of interventions.

A recurrent question around how stories could lead to change revisited the need to not just create stories, but also to amplify them such that they would be heard and acted upon by actors beyond the community—particularly policy-makers—to bring about changes in health equity outcomes.

How do we get these stories heard and out in the community?

What do we have to do to get these stories acted upon?

Reviewing the need for sustained co-production, participants explored ways community organisations could be linked with health professionals and creatives to undertake collective action. Participants discussed a “Community Innovation Team” model that had been introduced in Liverpool during the COVID-19 pandemic as a way of formalising such partnerships [21].

Building on the “preventative, positive, and long-term focused” theme from Workshop 3, participants narrowed down their ideal focus for future work to be around themes of:

Storytelling for promotion of health and wellbeing and prevention of ill health … stories of children and young people/parents; Childhood immunisations, breastfeeding and mental health; Focusing on ‘good news stories’ and how we can amplify stories; Using young people as a health education route into families; Focusing on least well-served communities; Access [to care] as a cross-cutting theme.

### Workshop 5: Programme design

Workshop 5 gathered views and visions of a research programme, discussing models of collaborative community partnerships and how to sustain these. We used a visioning exercise where participants were provided with a hypothetical future scenario where the consortium had obtained a research grant and completed three years of research to develop and test a creative health approach for Liverpool. They were then asked to imagine what four different people (a creative sector representative, public health commissioner, member of a disadvantaged community, and ReCITE consortium member) in this scenario said about the impact of the research on the community, arts, and health sectors, and what elements made the project successful. In this exercise, participants were able to use their own lived experiences to inform and illustrate their contributions of what success should look like.

Cross-cutting elements in participants’ visions of success included:

1) Empowerment, where people feel their stories are heard, the health system is accountable to them, they are taken seriously as agents for change, and they are given power to tackle health equity their way;

Communities [becoming] major drivers in their own health stories leading to accountability in the health sector.

2) Partnership, where different sectors embrace collaboration;

Arts organisations [becoming] active partners in the promotion of health stories and supporting health equity in the Liverpool region.

3) Evidence that leads to sustained funding, where different actors can take part in evaluation of impact on health equity and health organisations can draw on robust evidence showing measurable improvements to health equity.

I’ve known the importance of the arts for some time, but now I have some robust evidence of their centrality in effecting health equity.

Participants’ articulation of the elements that would underpin success were linked to the values expressed for the consortium in Workshop 1 particularly inclusivity and connectivity, meaningful collaboration and social justice. Participants spoke about working across disciplines especially between health and arts, connecting communities with policy and government apparatuses, and respecting different experiences.

I no longer feel isolated and powerless; by connecting with others, I can see routes for effecting change for and with the people I support.

They emphasised empowering communities, enriching relationships among partners, and sustained, long-term engagement with communities.

*[Success comes from] “creating a community where researchers*,* community groups and individuals work together to drive health equity in the area.”*

Finally, they highlighted the importance of dismantling hierarchies of power, creating space for genuine dialogue and critical reflection, and achieving organisational change and radical transformations.

Everyone [should] feel they have value. No one person [is] the ‘expert’.

### Workshop 6: Research questions

Workshop 6 condensed input from earlier workshops into research questions the consortium would address. While many aspects had been consolidated in Workshop 4 and 5, additional discussions returned to themes from Workshops 1–3. For example, participants picked up earlier discussions around the impacts of short-term funding cycles for community-led creative health projects and what evidence commissioners needed to fund these more sustainably, leading to refinement of a research question that would tackle this issue.

*“What can we do to promote closer alignment between communities and health commissioners on how funding decisions are made. How to close the gap and develop the middle ground?”*

Workshop 6 had a concrete aim of turning the final co-produced research programme into a funding application. This was submitted a few months after the conclusion of the workshop series and successfully received funding to begin carrying out the research programme in February of the subsequent year.

## ReCITE’s theory of change

**Fig. 2:**
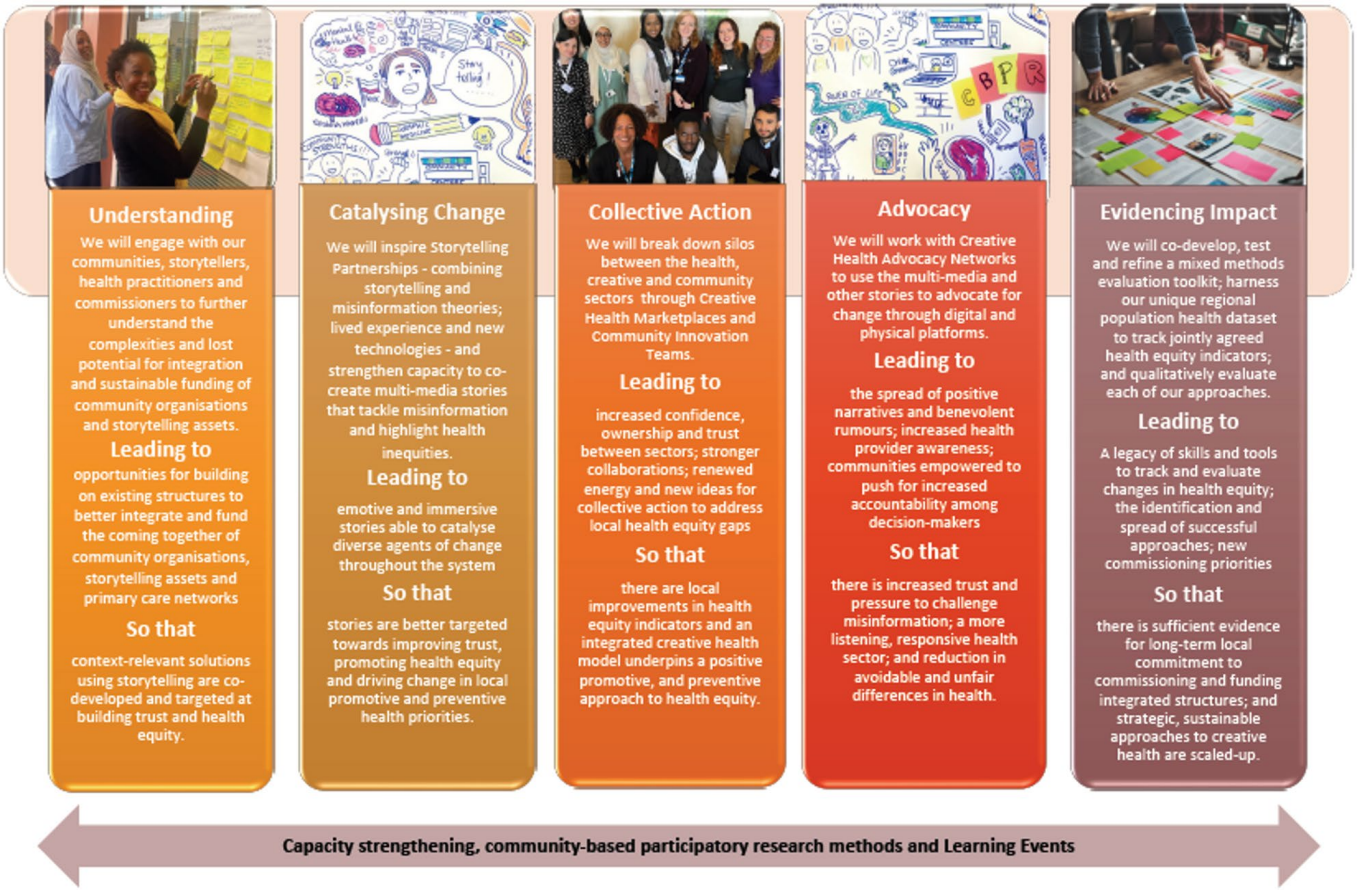
The co-produced theory of change for a creative health approach

In this section, we look at how inputs and outputs of the workshops shaped the development of ReCITE’s theory of change. A theory of change is a logic model that explains how a proposed approach (in our case, a community-led creative health approach based on storytelling) will create the desired outcome [22]. Each element in ReCITE’s theory of change reflects a recurring theme that emerged across the workshops, outlined in the previous section. The theory of change also embeds values important to members of the consortium, identified through a discussion in Workshop 1. Our theory of change is presented in two ways that reflect the needs and perspectives of different groups. In Fig. 2, a traditional logic model diagram depicts the theoretical pathway explaining the logic behind five research and intervention “pillars” that would lead to the desired outcome: sustained integration of storytelling into community and health systems to address health inequity. This format supported academic use of the theory of change, specifically to apply for research funding. Cross-cutting the five pillars are elements embedded across activities to empower communities, ensure non-academic partners have equitable involvement in research and intervention decisions, and share ongoing experiences.

**Fig. 3:**
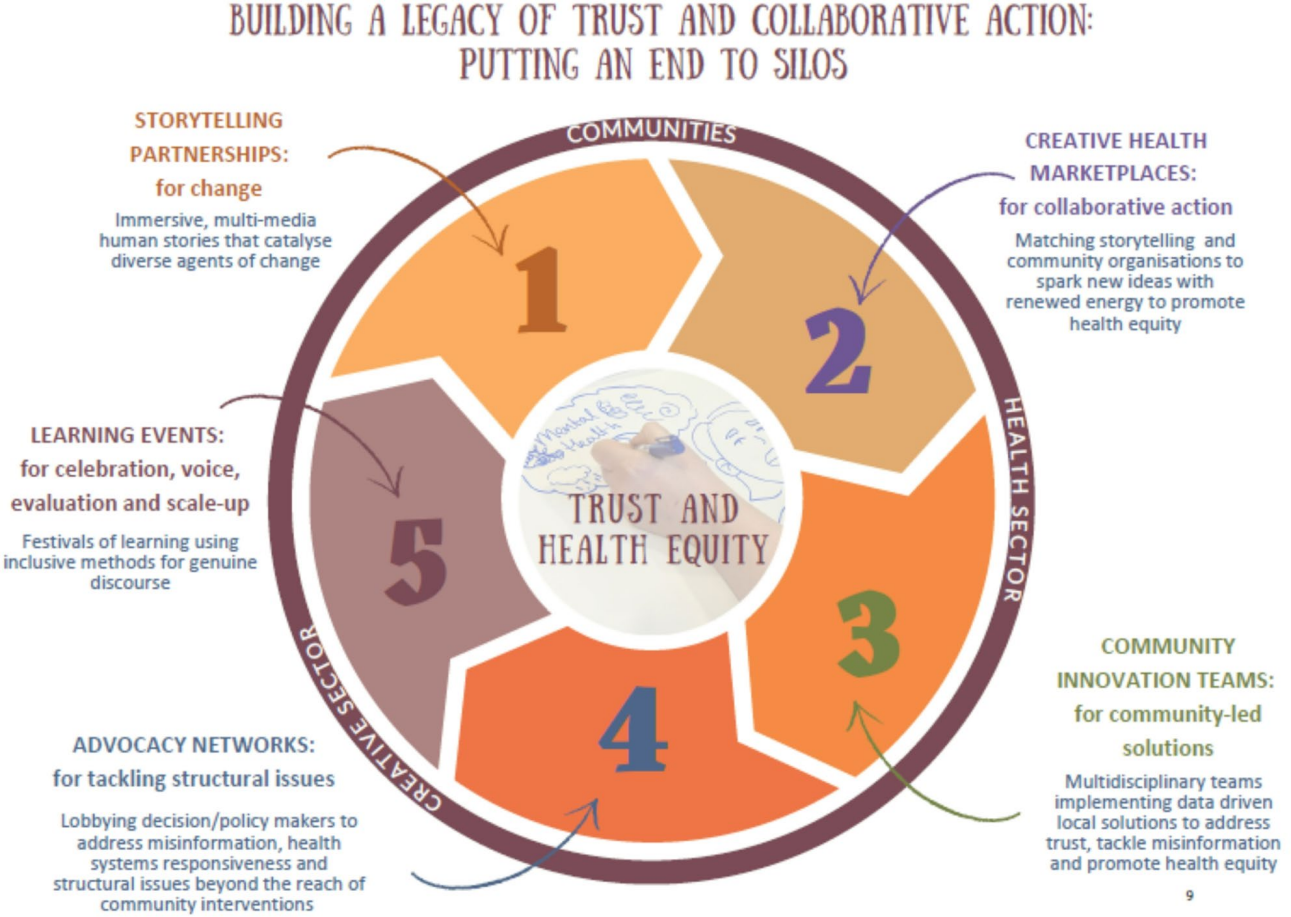
The wheel of creative health interventions to investigate in the co-produced research programme

An alternative depiction of the theory of change is illustrated with the intervention “wheel” in Fig. 3, which shows the interconnected activities that would drive the creative health approach forward to create systemic change. This action-focused design was particularly relevant for the non-academic stakeholders, as it put greater emphasis on the different sectors and their roles.

In this analysis, we focus on the theory of change as one output that arose out of the co-production activities, reflecting the themes of discussion and the consortium’s values. The theory of change is one of many outputs that came out of the co-production cycle (see Fig. 1; Table 1 for examples). It is important to note that although we analyse the co-production through the lens of the theory of change, all the outputs helped shape each other, later discussions, and indeed the composition and activities of the consortium itself.

### Pillar 1: Understanding the complexities in funding creative health

This research pillar seeks to understand the complexities and lost potential for integration and sustainable funding of community organisations and storytelling assets, as a foundation to develop solutions. It takes forward the discussion theme around existing knowledge held by communities and arts organisations and the potential for storytelling to advocate for health equity. Participants in workshops 1–3 expressed the impetus to better understand the impacts of short-term funding and the resultant missed opportunities for evidencing creative health approaches. This pillar thus focused on understanding the complexities involved in funding creative health.

### Pillar 2: Storytelling that catalyses change

The power of storytelling as a creative route for shaping attitudes and mobilising people was a key theme. This was not always positive. Stories could be used to reinforce existing perceptions and stereotypes, for example that unequal treatment or outcomes for a community were fixed, thereby entrenching inequity further. Participants recognised that there were problematic uses of storytelling as well, for example in spreading misinformation or sowing distrust and division. However, participants felt storytelling could make a difference to health equity by spotlighting health equity gaps and by creating conversations about health equity that individuals could better relate to and participate in. The workshop discussions reflected a need to focus on how stories could be used to catalyse action and lead to empowerment for different groups, rather than simply discuss existing narratives of unfair health disadvantages. This pillar was thus conceptualised as change-focused storytelling, directed at encouraging individuals to move beyond sharing experiences to working together to change them.

### Pillar 3: Achieving change through collective action

Throughout the workshops, ReCITE members and stakeholder groups reiterated the need to “break down silos” to achieve change at scale. Early in the series, participants discussed the need to link sectors and consolidate small projects to scale-up the impact storytelling could have. This informed the stakeholder mapping exercise, which in turn shaped the consortium itself and later workshops as a wider range of individuals were invited, for example, to Workshop 3 where a powerful consensus for collective action that was “preventative, positive, and long-term focused” emerged.

The pillar of collective action reflects the identified need for intersectoral collaboration to move beyond individual impact towards larger-scale change for health equity, harnessing the potential of the Community Innovation Team CIT) model proposed in Workshop 4 and already rolled out in Liverpool. Participants saw it as building on momentum and inspiration from individual and community stories to creating commitment for collaborative working to tackle agreed health priorities.

### Pillar 4: Advocacy to change societal structures

While the consortium identified collective action across sectors as a critical piece to produce change at scale, they also recognised that, in line with the consortium’s commitment to social justice, unfair societal structures needed to change—particularly those that perpetuate poverty. Poverty has an unequal impact on mental and physical health and drives complex community care putting pressure on health systems. The need to advocate for deeper structural change, rather than only tackling symptoms of inequality, shaped the development of this advocacy pillar as a crucial step to amplify stories to be heard and acted upon by higher layer of the system (commissioners, politicians and others) to bring about changes in health equity outcomes. Participants wanted to see how storytelling could help advocate for changes beyond what communities could themselves do, by creating compelling narratives that put pressure on decision-makers to enact structural change.

### Pillar 5: Evidencing impacts of a storytelling approach

A critical aspect of sustaining a creative health approach is how activities are funded. While participants with commissioning roles were concerned about justifying their decisions with robust evidence, participants from the creative and community sectors repeatedly questioned how and what evidence was considered robust enough to justify funding and commissioning decisions. This tension parallels one that we see in the literature on arts and health as well, where a wider academic debate exists over whether the current state of research provides sufficient evidence for creative health commissioning (see [11, 12] and critiques [13, 23]). These questions around evidence needs shaped a vision of the research programme as one that should provide qualitative and quantitative evidence on measurable health outcomes but also wider societal impacts. By providing multiple forms of evidence, the “evidencing impact” research pillar sought to bridge the gap between communities’ and commissioners’ needs and co-develop tools and indicators that different sectors could contribute to.

## Discussion

ReCITE’s Theory of Change provides a model for how creative health can be used as a community-led approach to improve health equity. It uses storytelling as a mechanism to affect change to structural drivers of health inequity. Both the discussions around the reported literature (workshop 2) and subsequent sharing of experiences from diverse stakeholder groups highlighted a need to go beyond research targeting individual experiences and outcomes and instead understand how to intervene at the population and systems level. Stakeholders gave input and reviewed the model through the workshops, with consortium members taking the lead to consolidate it and acquire funding for a research programme to investigate the model. The research programme was successfully funded, and the project started eight months after the conclusion of the workshop series, with an expanded ReCITE consortium leading it. The workshops were thus successful in achieving their goals to build a research-ready consortium and co-produce (and get funding for) a research programme based on a theory of change for creative health. In this discussion, we reflect on how well the iterative co-production process enabled input from different stakeholders into these outputs and how that reflected power held by different stakeholders to emphasise their priorities and benefit from outcomes of the work.

### Whose input goes into the co-production spiral?

The ReCITE consortium-building process was an iterative one where cross-sectoral representation and input was sought over a sustained period to grow the consortium while generating multiple outputs that fed into subsequent products (see Fig. 1). Sustained engagement was necessary to develop the common understanding and trust, not just to inform the research programme but also establish the research-readiness of the consortium (see, e.g., [24]). The workshop series provided the structure for stakeholders to build trust over repeated engagement, and also allowed for greater inclusion into the research design: stakeholders who did not have time to commit to the full process could give input, for example in workshop 3, which prioritised hearing from arts, health, and community groups, and received the highest participation from these groups. Stakeholders who were not initially aware of the project were also welcomed at later stages to help shape the research programme. An organisation within each sector was able to send a representative to at least half the workshops, while the academic, creative, and capacity development sectors had an organisation that was represented at all six workshops.

While flexibility in participation increased opportunity to be included in the co-production, it also meant that levels of engagement varied from one-off contributions (a lower level of involvement that is arguably not considered co-production) to repeated involvement and review of earlier outputs (part of the co-production process to shape the development of outputs), to making final decisions on outputs (full co-production, with power to determine an output’s final form). Inevitably stakeholders who could commit the time to attend more workshops were better represented in decision-making. Repeat attendance tended to be highest from the original partners who had received the initial funding to run the workshops and therefore held responsibility for programme outcomes. Programme ownership and dedicated funding thus appeared to be facilitators of engagement. Even so, only six individuals attended all six workshops, underscoring the difficulty of committing time to every part of a lengthy process. Time and the lack of funding to support that time commitment was a barrier for stakeholders from the community and health sectors. Even though many organisations were keen to be included, they reported being overstretched for time and capacity to send representatives.

As a strategy to overcome inclusion barriers while ensuring the work moved forward, the workshops interspersed phases of *consultation* and *consolidation*. This meant that stakeholder input in an initial workshop was consolidated during a subsequent workshop and fed back into further consultation in an iterative process. It is difficult to determine the extent to which any single stakeholder co-produced later stage outputs, but in general, stakeholders who attended more than one workshop would have given feedback on earlier contributions. For example, this paper is itself co-produced by the authors (representing sectors noted in our positionality statement); it draws on and quotes workshops led by a different set of third sector and creative partners (but supported by academic partners). The theory of change involved consultative input from some stakeholders and decision-making input from others over the whole process.

Nonetheless, lower representation from community and health sectors would have impacted their ability to influence decision-making and prioritisation of issues, since they were underrepresented relative to other sectors at the consolidation workshops. Of course, classifying in sectors based on their primary affiliations, as we have done in this paper, masks the fact that many participants held dual affiliations and have themselves personal or professional lived experiences that cross-cut sectors. For example, our arts and community sector representatives often had cross-over in their work. However, while this helped incorporate perspectives from the different sectors into project outputs, many organisations who were stretched for capacity and time and thus struggled to sustain attendance at workshops would still be underrepresented in decision-making.

### How can co-production outputs reflect the priorities of different partners?

Our co-production work resulted in multiple outputs over the course of the workshop, with examples shown in Table 1. We have analysed in this paper how perspectives and priorities of different sectors (arts, community, health, academic) informed the underpinning theory of change and the direction of inquiry for this research programme. However, an interesting further question is whether individual partners’ priorities—which to some extent motivated their participation—were also reflected in the outputs. One key priority for most stakeholders was to achieve longer-term sustainability both for the overall programme of work they had co-produced and for their involvement in it. This is an ongoing concern within a landscape of underfunding for the arts and community sectors and lack of incentives for health providers to explore creative health approaches [25], which leaves organisations competing for small pots of funding. Research funding for co-production is also limited and rarely sufficient to support overstretched public and third sector organisations to commit time to participate. The ReCITE project was already unusual in receiving funding from the beginning to support PPIEP activities, including for a few non-academic partners to participate over the nine-month time period. Even so, there was more interest in the project from stakeholders than it was possible to fund. The workshop discussions suggest that lack of funding constrains ongoing participation in co-production, especially from smaller organisations and health providers who are less able to commit staff time.

Without ongoing funding support, the level of time and effort each partner is willing to place on certain outputs can be driven by the value of that output within the relevant sector [26]. Some of our stakeholders joined our workshops with hopes of shared financial support, and this motivated a specific research question around understanding how funding for community-led creative health interventions can be sustained. The consortium subsequently successfully acquired research programme funding with an application that included funds for community and health organisations. However, those funds still could not include every individual stakeholder, given the number of stakeholders who wanted to be involved, restrictions from funders about how much funding could be allocated to non-academic stakeholders, and a need for proposed activities to fit the funder’s scope. This created tension when stakeholders’ individual priorities were not just to have their perspectives incorporated in outputs, but to secure future funding for their activities.

ReCITE’s iterative methodology provided a way to overcome some of the barriers to research inclusion, by offering participation flexibility and inclusivity over time. However, funding continues to be the critical challenge for conducting genuine, long-term, and iterative co-production in research. Piecemeal and short-term funding for participating stakeholders risks the sustainability of ongoing co-production. The temptation to maximise funding application success can also create a disconnect between what stakeholders need and what research funders will find appealing. Meaningful participation needs to be grounded in trusted relationships that require time to develop. Without adequate compensation for all non-academic stakeholders and clear communication around expectations, trust can be undermined, reducing engagement and potentially leading to perceptions of tokenism [27]. Addressing this issue may require a radical rethink of how funding is allocated for co-producing research and who is contracted to deliver co-production activities—should this be academic or non-academic organisations? This question relates to a structural issue that our stakeholders identified: who determines the evidential standards for funding, what outputs are of value to funders, and which stakeholders holds power at that table?

## Conclusion

This paper offers two main contributions to the literature. First, a co-produced theory of change that (1) addresses a gap in the extant literature on creative health regarding its potential impact on health equity and the social determinants of health, and (2) places community at the heart of the model to lead interventions. This provides a logic model that can be evaluated in future research to provide needed evidence on the impact of pairing community-led and creative health approaches.

Second, the process of co-producing that theory of change (along with multiple other outputs, including a unique creative health research programme) demonstrates a case study of complex public involvement across multiple sectors with a stake in health equity. Our iterative process of consultation and consolidation resulted in multiple outputs that fed into a systems-level approach towards researching community-led creative health for health equity, not just focusing on the impact of interventions but how they can be sustained in the longer-term. Importantly, the process has not concluded with the workshops but evolved into a longer-term partnership with stakeholders across sectors that is still continuing at time of writing. This model for building and sustaining partnerships over time relied on longer-term relationship building among different stakeholders to allow their priorities to be captured and acted upon. It offers several lessons for longer-term participatory research projects (and funders of these). To sustain genuine, longer-term, and iterative co-production, partners need to be prepared for the time commitment involved, and funders need to be willing and able to fund a longer-term process with that builds trust among partners and supports equitable power-sharing in decision-making and project priorities.

## Supplementary Information

Below is the link to the electronic supplementary material.

Supplementary Material 1

## Data Availability

Data (in the form of workshop reports) and materials are available from the authors on request.

## Footnotes

1 The review was undertaken by research partner, The Stop Collaborate and Listen Agency, as an internal scoping review of evidence available on the impact of art, culture and storytelling on health and wellbeing inequity, and the existing mechanisms, methods, and models that attempt to evaluate the connection between these. A private report of this work was made to the consortium. A summary of the report is provided in the Appendix.

## Declarations

### The ReCITE consortium (2022–2023) is led by the Liverpool School of Tropical Medicine and consists of

Miriam Taegtmeyer (Liverpool School of Tropical Medicine); Rachel Tolhurst (Liverpool School of Tropical Medicine); Gillian Kyalo (Liverpool School of Tropical Medicine); Amina Ismail (Liverpool School of Tropical Medicine); Mike Morris (Writing on the Wall); Madeline Heneghan (Writing on the Wall); Natalie Denny (Writing on the Wall); Amanda Fulford (Edge Hill University); Sarah Maclennan (Liverpool John Moores University) Victoria I. Ekpo (Edge Hill University); Vicki Doyle (Capacity Development International); Ema Kelly (Capacity Development International); Kim Ozano (The Stop, Collaborate and Listen Agency Ltd); Sarah Glover (The Stop, Collaborate and Listen Agency Ltd).

## References

[1] Lang I, King A, Jenkins G, Boddy K, Khan Z, Liabo K. How common is patient and public involvement (PPI)? Cross-sectional analysis of frequency of PPI reporting in health research papers and associations with methods, funding sources and other factors. BMJ Open. 2022;12:e063356. 35613748 10.1136/bmjopen-2022-063356 PMC9131100

[2] Bergholtz J, Wolf A, Crine V, Cleeve H, Santana M-J, Björkman I. Patient and public involvement in healthcare: A systematic mapping review of systematic reviews - identification of current research and possible directions for future research. BMJ Open. 2024;14:e083215. 39304210 10.1136/bmjopen-2023-083215 PMC11418490

[3] Smith H, Budworth L, Grindey C, Hague I, Hamer N, Kislov R, et al. Co-production practice and future research priorities in united Kingdom-funded applied health research: a scoping review. Health Res Policy Syst. 2022;20:36. 35366898 10.1186/s12961-022-00838-x PMC8976994

[4] International Association for Public Participation. Public participation pillars. https://www.iap2.org/page/pillars. (Accessed 1 October 2025).

[5] Ozano K, Alam W, Aktar B, Okoth L, Chumo I, Quach JA, et al. Seven core competencies and conditions for equitable partnerships and power sharing in community-based participatory research. BMJ Global Health. 2024;9:e015497. 39551576 10.1136/bmjgh-2024-015497 PMC11574420

[6] Filipe A, Renedo A, Marston C. The co-production of what? Knowledge, values, and social relations in health care. PLoS Biol. 2017;15:e2001403. 28467412 10.1371/journal.pbio.2001403 PMC5414996

[7] The All-Party Parliamentary Group on Arts, Health and Wellbeing and the National Centre for Creative Health. Creative Health Rev How Policy Can Embrace Creative Health. 2023. https://ncch.org.uk/creative-health-review.

[8] Agnello DM, Anand-Kumar V, An Q, de Boer J, Delfmann LR, Longworth GR, et al. Co-creation methods for public health research — characteristics, benefits, and challenges: a health CASCADE scoping review. BMC Med Res Methodol. 2025;25:60. 40050729 10.1186/s12874-025-02514-4 PMC11884017

[9] MacGregor S, Cooper A, Searle M, Kukkonen T. Co-production and arts-informed inquiry as creative power for knowledge mobilisation. Evid Policy. 2022.

[10] Langley J, Kayes N, Gwilt I, Snelgrove-Clarke E, Smith S, Craig C. Exploring the value and role of creative practices in research co-production. Evid /& Policy. 2022.

[11] Fancourt D, Finn S. What is the evidence on the role of the arts in improving health and well-being? A scoping review. WHO Regional Office for Europe: Copenhagen. 2019. http://www.ncbi.nlm.nih.gov/books/NBK553773/. (Accessed 30 Jun 2025). 32091683

[12] Fancourt D, Warran K, Aughterson H. Evidence summary for policy. The role of arts in improving health & wellbeing. Report to the Department for Digital, Culture, Media & Sport. 2020. https://assets.publishing.service.gov.uk/media/5f9812268fa8f543f786b37f/DCMS_report_April_2020_finalx__1_.pdf.

[13] Grebosz-Haring K, Clift S. The need for a critical perspective on arts and health research and evidence reviews. In Routledge Handbook of Arts and Health. 2025. 10.1016/j.plrev.2025.07.005 40644828

[14] Ashton M. State of health in the city: Liverpool 2040. 2024. https://liverpool.gov.uk/council/public-health-liverpool/state-of-health-in-the-city/

[15] Botfield JR, Newman CE, Lenette C, Albury K, Zwi AB. Using digital storytelling to promote the sexual health and well-being of migrant and refugee young people: A scoping review. Health Educ J. 2018;77:735–48.

[16] Fadlallah R, El-Jardali F, Nomier M, Hemadi N, Arif K, Langlois EV, et al. Using narratives to impact health policy-making: A systematic review. Health Res Policy Syst. 2019;17:26. 30836972 10.1186/s12961-019-0423-4 PMC6402129

[17] de Jager A, Fogarty A, Tewson A, Lenette C, Boydell KM. Digital storytelling in research: A systematic review. Qualitative Rep. 2017;10:2548–82.

[18] Ings R, McMahon J. Arts and culture in health and wellbeing and in the criminal justice system: a summary of evidence. Arts Council Engl. 2018. https://www.artscouncil.org.uk/arts-and-culture-health-and-wellbeing-and-criminal-justice-system-summary-evidence.

[19] Rutter H, Savona N, Glonti K, Bibby J, Cummins S, Finegood DT, et al. The need for a complex systems model of evidence for public health. Lancet. 2017;390. 10.1016/S0140-6736(17)31267-9. 10.1016/S0140-6736(17)31267-9 28622953

[20] Stickley T, Parr H, Atkinson S, Daykin N, Clift S, De Nora T, et al. Arts, health & wellbeing: reflections on a National seminar series and Building a UK research network. Arts Health. 2017;9:14–25. 28163778 10.1080/17533015.2016.1166142 PMC5215041

[21] Infection Innovation Consortium. The Health Equity Liverpool Project (HELP). 2024. https://www.infectioninnovation.com/case-studies/the-health-equity-liverpool-project-help/.

[22] Breuer E, Lee L, De Silva M, Lund C. Using theory of change to design and evaluate public health interventions: A systematic review. Implement Sci. 2016;11:63. 27153985 10.1186/s13012-016-0422-6 PMC4859947

[23] Clift S, Phillips K, Pritchard S. The need for robust critique of research on social and health impacts of the arts. Cult Trends. 2021;30(5):442–59. 10.1080/09548963.2021.1910492.

[24] de Montigny JG, Desjardins S, Bouchard L. The fundamentals of cross-sector collaboration for social change to promote population health. Global Health Promotion. 2019;26:41–50. 28805502 10.1177/1757975917714036

[25] Ederer F, Manso G. Is Pay-For-Performance Detrimental to Innovation?. 2009. https://escholarship.org/uc/item/03t787q9. (Accessed 30 Jun2025).

[26] Oliver K, Kothari A, Mays N. The dark side of coproduction: do the costs outweigh the benefits for health research? Health Res Policy Syst. 2019;17:33. 30922339 10.1186/s12961-019-0432-3 PMC6437844

[27] Gilchrist K, Iqbal S, Vindrola-Padros C. The role of patient and public involvement in rapid qualitative studies: can we carry out meaningful PPIE with time pressures? Res Involv Engagem. 2022;8:67. 36451246 10.1186/s40900-022-00402-5 PMC9713187

