# Supplementary material for: Patient and public involvement, engagement, and participation in practice: co-production of a creative health approach and theory of change through the ReCITE consortium-building project in Liverpool
Source: Res Involv Engagem. 2026 Feb 26;12:26. doi: 10.1186/s40900-026-00846-z (PMC12937545; doi:10.1186/s40900-026-00846-z)
Supplement: Supplementary file 1 — Supplementary Material 1 [file 40900_2026_846_MOESM1_ESM.docx]

**Appendix**

**Summary of internal report for literature review (Workshop 2)**

The key findings from this scoping review indicated that storytelling was an especially effective method for engaging marginalised, vulnerable, and disadvantaged groups. It was commonly utilised to enhance education and training among healthcare professionals, promote positive mental health, support recovery, and challenge dominant, oppressive narratives surrounding issues related to race, disability, sexuality, and other social inequities. Furthermore, storytelling served as a means to address colonial legacies through culturally grounded practices and as a powerful tool for advocacy and policy change aimed at combating health inequities. However, the review also underscored significant ethical considerations when using storytelling with individuals who had experienced trauma.

Regarding the measurement of impact, the review found that while storytelling showed promise, there was a notable lack of robust, standardised, and long-term evaluations assessing its effects on health inequities. Current measures have predominantly focused on short-term changes in knowledge, attitudes, or perceptions rather than sustained outcomes considered from an equity perspective. Therefore, it was recommended that there be a stronger theoretical foundation regarding how storytelling and arts-based approaches contributed to reducing health inequities, coupled with increased investment in evaluation capacity and funding infrastructure to facilitate rigorous, long-term research and scaling up within the third sector.

This scoping review also highlighted the value of applying a social ecology of power framework to evaluate how storytelling impacts health inequities across micro (individual), meso (institutional), and macro (societal) levels. While storytelling provides important individual benefits such as empowerment, connection, and wellbeing, the review highlighted the importance of moving beyond the individual level to understand and address the broader institutional and structural factors that shape health inequities. At the meso level, storytelling can shift power dynamics, improve access for marginalised groups, influence professional attitudes, and drive advocacy and policy change. At the macro level, it challenges colonial and oppressive narratives, preserves cultural heritage, and promotes anti-racism and social justice. Overall, this multilevel perspective highlights storytelling’s potential as a transformative practice for addressing health inequities, ranging from personal healing to systemic and societal change.
